# Diet selection and n-3 polyunsaturated fatty acid deposition in lambs as affected by restricted time at pasture

**DOI:** 10.1038/s41598-017-15875-8

**Published:** 2017-11-15

**Authors:** X. Q. Zhang, Y. M. Jin, W. B. Badgery, Tana

**Affiliations:** 1 Institute of Grassland Research, CAAS, Hohhot, 010010 China; 2 Marine college, Shandong University at Weihai, Weihai, 264209 China; 3 New South Wales Department of Primary Industries, Orange Agricultural Institute, Orange, NSW 2800 Australia

## Abstract

This study investigates the effects of restricted grazing time on forage selectivity and meat fatty acid deposition of lambs compared to a traditional grazing system. Results showed that the animals preferred to graze pasture species that were more palatable and lower in fibre, while demonstrating a partial preference for species with high protein levels. *Leymus chinensis* was more preferentially selected by lambs grazing pasture for shorter periods than longer periods. Lambs that grazed for 4 h per day had a high LNA (α-linolenic acid) intake. The accumulation of LNA and its elongation products in tissue was closely related to the LNA level in diet. Therefore, allowing lambs to graze for at least 4 h per day resulted in a meat fatty acid profile that is richer in health-promoting fatty acids. In particular, the highest DHA (docosahexaenoic acid) was observed in meat from lambs which grazed pasture for 4-h versus 8- and 12-h. It can be concluded that, in grassland systems, a healthier meat fatty acid profile for humans and the potential for better pasture management is achieved by limiting the grazing of lambs to 4 h per day rather than grazing over longer time periods.

## Introduction

Long-chain n-3 polyunsaturated fatty acids (PUFA), including eicosapentaenoic acid (EPA, C20:5 n-3) and docosahexaenoic acid (DHA, C22:6 n-3), have been shown to enhance human health^1^. Meat, fish, fish oils and eggs are the only significant sources of long-chain n-3 PUFAs in human diets. Although meat has lower concentrations of these fatty acids compared to oily fish, it is a significant source for many people with low levels of fish in their diet.

Sheep meat can have beneficial fatty acid (FA) properties when grazed on pasture. However, continuous grazing on degraded grasslands results in poor animal performance that can, in-turn, reduce meat quality through lower fat content, or increased age of animals at slaughter. To maximize animal productivity and promote grassland restoration, there is renewed interest in restricting access to pasture in combination with indoor supplementation in pastoral livestock systems in China^2,3^. The restriction of grazing time is a way to budget limited pasture, while supplementing to produce high-quality lamb meat. Compared to meat from animals fed concentrates indoors, grazing pasture, even for restricted periods of time each day, improves the FA properties of lamb’s meat^4,5^ and cow’s milk^6^. The diurnal variation in the LNA (α-linolenic acid, C18:3 n-3) content of ryegrass (*Lolium Perenne*) in the sward and the time of day animals were given access to grazing was a factor influencing the FA composition of meat and milk. Meat from lambs restricted to 8-h grazing had increased healthy FAs compared to meat from lambs restricted to 2-h grazing on desert steppe due to their higher pasture intake^5^. To date, very little research has been conducted investigating how restricted grazing regimes alter diet selection (relative intake of individual forage species), and the consequent influence this has on the meat FA profile of lambs when they grazed on natural grasslands with abundant forage species.

The objective of this study was to investigate changes in lamb foraging selectivity in response to daily restrictions of time at pasture, and the effects of restricted grazing time on meat FA composition compared to a traditional grazing system in which animals were allowed to graze for the whole day. Additionally, the relationships between amounts of LNA and linoleic acid (LA, C18:2 n-6) consumed in the diet and the LNA level deposited in meat were assessed. We hypothesized that higher intake of LNA from *L. chinensis* will be found with greater restriction of grazing and that this will result in subsequent increases in healthier FAs of EPA and DHA in the diet and muscle composition.

## Results

### Feed chemical composition

Feed chemical composition was significantly affected by plant species, growing month and their interaction (Table 1). The highest (*P* < 0.001) metabolizable energy (ME) was found in concentrate; among-pasture species, it was higher (*P* < 0.001) in *Stipa krylovii* and *L. chinensis* compared to *Cleistogenes squarrosa* and *Allium ramosum*. The crude protein (CP) content was higher (*P* < 0.001) in *A. ramosum* than concentrate; and it was higher (*P* < 0.001) in *L. chinensis* than in *S. krylovii*, *C. squarrosa* and *Carex duriuscula*, respectively. The highest levels of neutral detergent fibre (NDF) were found in *S. krylovii* and *L. chinensis*, the lowest in *A. ramosum*, with moderate levels in *C. squarrosa* and *C. duriuscula*.

**Table 1:** Chemical composition of grass hay, concentrate and pasture from July to September.

Item	Forage species							s.e.m.	*P*-value^2^		
	*S. krylovii*	*C. squarrosa*	*L. chinensis*	*C. duriuscula*	*A. ramosum*	Grass hay^1^	Concentrate^1^		Species (S)	Month (M)	S × M
ME (MJ kg^−1^)	8.78^b^	7.59^c^	8.19^b^	7.93^bc^	6.26^d^	6.67^d^	12.30^a^	0.24	0.0002	0.011	0.034
CP (g kg^−1^ DM)	81^c^	90^c^	149^b^	92^c^	240^a^	59^d^	187^b^	1.15	<0.001	<0.001	<0.001
NDF (g kg^−1^ DM)	617^ab^	408^c^	590^b^	469^c^	214^d^	665^a^	27^e^	28	<0.001	<0.001	<0.001
C10:0 (g kg^−1^ DM)	0.08^b^	0.04^c^	0.01^e^	0.02^d^	0.47^a^	0.02^d^	0.01^e^	0.03	<0.001	<0.001	<0.001
C12:0 (g kg^−1^ DM)	0.25^c^	0.30^b^	0.17^d^	0.14^e^	0.49^a^	0.12^e^	0.02^f^	0.03	<0.001	<0.001	<0.001
C14:0 (g kg^−1^ DM)	0.32^c^	0.32^c^	0.71^b^	0.23^d^	1.17^a^	0.18^d^	0.11^d^	0.06	<0.001	<0.001	<0.001
C16:0 (g kg^−1^ DM)	6.55^e^	7.83^d^	8.66^d^	9.14^c^	22.26^a^	3.98^f^	13.19^b^	1.12	<0.001	<0.001	<0.001
C18:0 (g kg^−1^ DM)	0.68^d^	0.95^c^	0.67^d^	1.16^b^	3.24^a^	0.52^d^	1.60^b^	0.18	<0.001	<0.001	<0.001
C18:1cis-9 (g kg^−1^ DM)	0.84^c^	1.31^b^	0.49^d^	1.32^b^	0.80^c^	0.37^d^	14.10^a^	0.09	<0.001	<0.001	<0.001
C18:2 n-6 (g kg^−1^ DM)	4.33^d^	5.15^c^	5.13^c^	4.98^cd^	5.38^b^	1.74^e^	29.74^a^	0.18	<0.001	<0.001	<0.001
C18:3 n-3 (g kg^−1^ DM)	7.95^c^	7.65^c^	19.31^a^	10.30^b^	7.97^c^	3.25^d^	1.17^e^	1.20	<0.001	<0.001	<0.001
C20:3 n-3 (g kg^−1^ DM)	–^3^	—	—	—	0.26	—	—	0.02	/^4^	/	/
C20:5 n-3 (g kg^−1^ DM)	—	—	—	—	2.53	—	—	0.17	/	/	/

^a–f^Means within a row with different superscripts differ significantly (*P* < 0.05). ^1^The difference in chemical composition between forage species during July to September were analysed using repeated measures in the MIXED procedure, while the differences between concentrate and grass hay were examined using a two-sample t-test for means^2^ Probability values for main and interaction effects^3^– = undetectable^4^ / = no statistical analysis. ME = metabolizable energy; CP = crude protein; DM = dry matter; DNF = neutral detergent fibre; s.e.m. = standard error of mean.

For the FA composition, *A. ramosum* contained the greatest (*P* < 0.001) amounts of C10:0, C12:0, C14:0, C16:0 and C18:0 among plant species, while concentrate had higher (*P* < 0.001) C18:1 cis-9 and LA contents compared to other feedstuffs (Table 1). The content of LNA was higher (*P* < 0.001) in *L. chinensis* than other plants. C20:3 n-3 and EPA were found in *A. ramosum* but they were not measured in other forage species.

### Foraging diet composition

**Figure 1:**
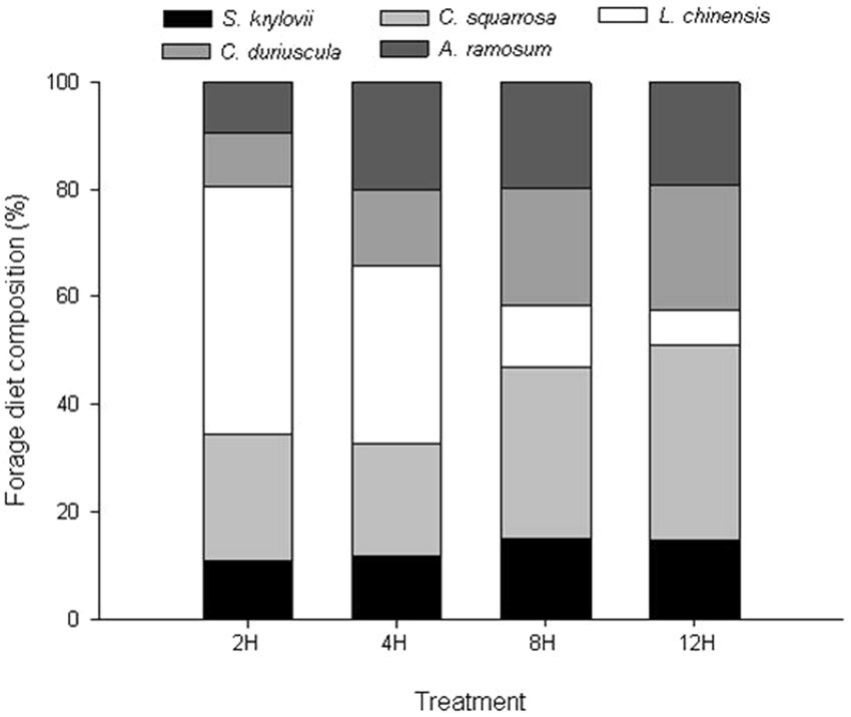
Composition of foraging species consumed by lambs. The lambs allocated to 2-h (2H), 4-h (4H), 8-h (8H) and 12-h (12H) grazing on the typical steppe in each month from July to September period.

Clear between-month differences in the proportions of various dietary components were observed for the four treatment diets (Fig. 1). For all months, the forage diet for 12H treatment was made up of 36% *C. squarrosa*, 24% *C. duriuscula*, 19% *A. ramosum*, 15% *S. krylovii* and 6% *L. chinensis*; for 8H treatment those were accordingly 32%, 22%, 20%, 15% and 11%, respectively (Fig. 1). The relative contributions of *C. squarrosa* and *C. duriuscula* to the 8H and 12H treatments were higher than that to the 2H and 4H treatments. When time allowed at pasture was reduced from 12 to 2H, the consumptions of *L. chinensis* increased from 6% with 12H to 33–46% with 2H or 4H of access.

**Figure 2:**
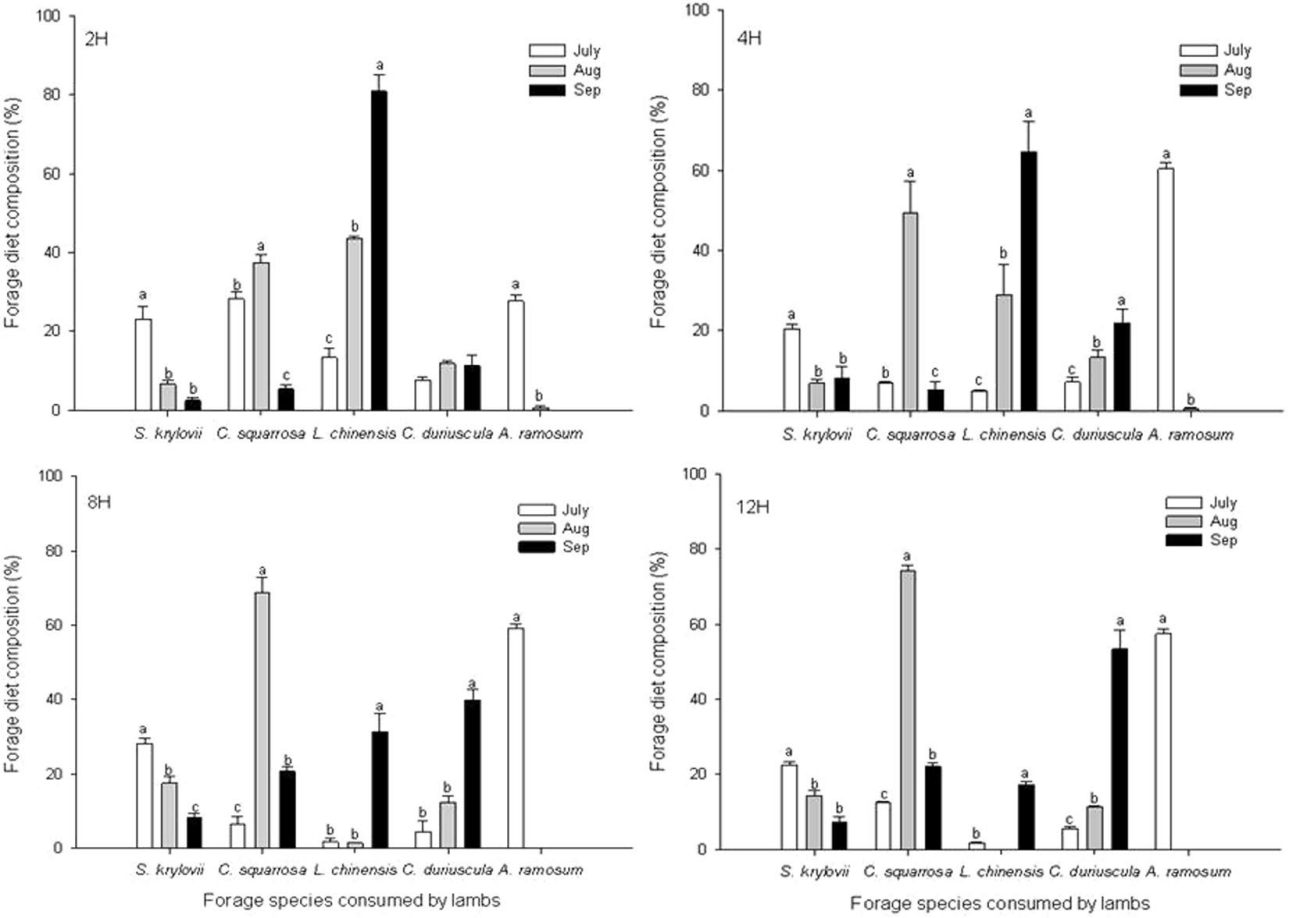
Forage diet composition of lambs. The lambs allocated to 2-h, 4-h, 8-h and 12-h grazing on the typical steppe over grazing periods from July to September period. Significant differences (*P* < 0.05) are represented by different letters. Error bars are a standard error of mean (s.e.m.).

The increase in *L. chinensis* in the diet of lambs grazing in the 2H and 4H treatments mainly occurred due to increasing consumption of this species as the grazing season progressed (Fig. 2), which did not occur in the longer grazing treatments. Its consumption decreased from September, to August with the least in July; while the peak use of *C. squarrosa* and *C. duriuscula* was in August and September, respectively. *S. krylovii* was a minor part of the four forage diets over the three months, with the highest consumption in July (Fig. 2). In addition, an increased relative intake of *A. ramosum* was observed in 4H, 8H and 12H treatments compared to the 2H treatment (+10%), making it one of the main dietary components in the former three treatments. Its consumption dramatically declined from July to August, and disappeared from diet and pasture in September.

**Figure 3:**
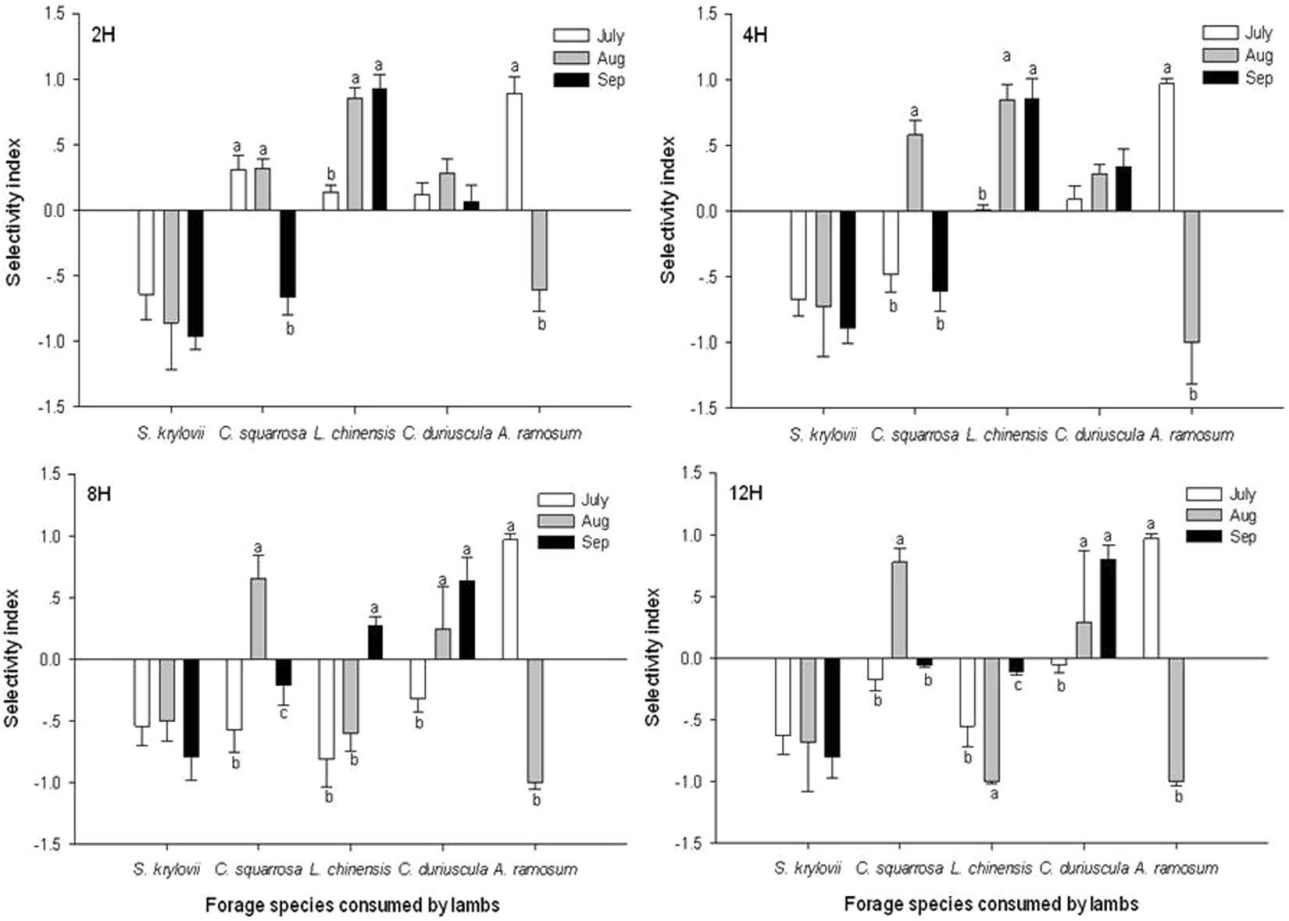
Selectivity index of lambs. The lambs allocated to 2-h, 4-h, 8-h and 12-h grazing on the typical steppe in each month from July to September period. Significant differences (*P* < 0.05) are represented by different letters. Error bars are a standard error of mean (s.e.m.).

The selectivity index for each forage species changed over grazing months (Fig. 3). The selectivity index for *S. krylovii* showed a similar pattern in all four treatments; it was avoided by the lambs to similar degrees in each month. The selection for *C. squarrosa* was avoided to varying degrees by all lambs in September, but was positively selected by the 2H lambs in July-August and 4H, 8H and 12H lambs in August. Lambs in 2H and 4H treatments showed stronger selection for *L. chinensis* than those in 8H and 12H treatments over the grazing period. The selectivity index for *C. duriuscula* was positively selected by lambs in the 2H and 4H treatments during all the grazing months, but in the 8H and 12H lambs it was positively selected in the latter two months. *A. ramosum* was preferred by the lambs in July but was avoided in August and not present in September.

### FA intake

FA intake was affected by treatment and month (Table 2). The average intakes of C10:0 (*P* = 0.001), C12:0 (*P* = 0.0001), C14:0 (*P* = 0.002) and C18:0 (*P* = 0.036), LNA (*P* = 0.014), C20:3 n-3 (*P* = 0.006) and EPA (*P* = 0.003) were higher for 4–12 h grazed lambs than 2 h grazed lambs, and there were no significant differences between 4–12 h grazed treatments. C16:0 intake was higher (*P* = 0.0002) in the 4H treatment than the other three treatments. C18:1 cis-9 intake significantly decreased (*P* < 0.001) as the time at pasture increased; whereas the largest (*P* < 0.001) intake of C18:2 n-6 was in the 2H treatment, the smallest in the 12H treatment, and the moderate level in 4H and 8H treatments.

**Table 2:** Fatty acid intake of lambs restricted to 2-, 4-, 8-, and 12-h access to pasture.

Item	Treatment^1^				s.e.m.	*P*-value^2^		
	2H	4H	8H	12H		Trt (T)	Month (M)	T × M
C10:0 (g day^−1^ DM)	0.06^b^	0.14^a^	0.14^a^	0.15^a^	0.03	0.001	<0.001	0.005
C12:0 (g day^−1^ DM)	0.20^b^	0.31^a^	0.33^a^	0.37^a^	0.02	0.0001	<0.001	0.073
C14:0 (g day^−1^ DM)	0.45^b^	0.64^a^	0.57^a^	0.60^a^	0.04	0.002	<0.001	0.068
C16:0 (g day^−1^ DM)	13.10^b^	16.01^a^	15.31^ab^	14.95^ab^	1.05	0.0002	<0.001	0.095
C18:0 (g day^−1^ DM)	1.54^b^	2.02^a^	1.99^a^	1.97^a^	0.18	0.036	<0.001	0.041
C18:1cis-9 (g day^−1^ DM)	6.12 ^a^	4.67^b^	4.39^c^	3.27^d^	0.23	<0.001	<0.001	0.001
C18:2 n-6 (g day^−1^ DM)	15.31^a^	13.07^b^	12.36^b^	10.21^c^	0.55	<0.001	<0.001	0.014
C18:3 n-3 (g day^−1^ DM)	9.88^b^	12.34^a^	11.54^a^	12.84^a^	1.195	0.014	<0.001	0.052
C20:3 n-3 (g day^−1^ DM)	0.03^b^	0.08^a^	0.07^a^	0.07^a^	0.02	0.006	<0.001	0.001
C20:5 n-3 (g day^−1^ DM)	0.20^b^	0.62^a^	0.58^a^	0.57^a^	0.16	0.003	<0.001	0.001

^a–d^Means within a row with different superscripts differ significantly (*P* < 0.05). ^1^Treatments: 2H = 2-h access to pasture; 4H = 4-h access to pasture; 8H = 8-h access to pasture; 12H = 12-h access to pasture (control). ^2^Probability values for main and interaction effects. DM = dry matter; s.e.m. = standard error of mean.

### Muscle FA composition

**Figure 4:**
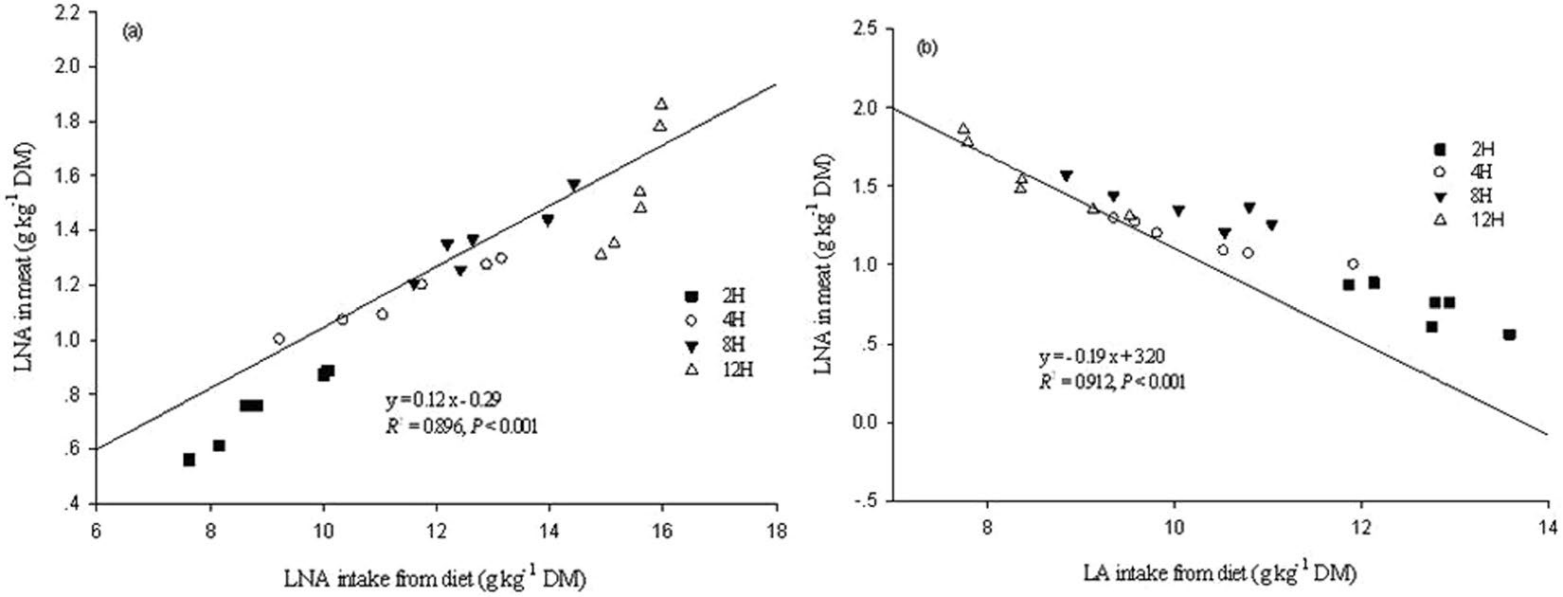
Relationships between amounts of LNA (**a**) and LA (**b**) intakes from diet and the LNA level deposited in lamb meat. LNA represents α-linolenic acid (C18:3 n-3), while LA represents linoleic acid (C18:2 n-6). DM is dry matter. The data used were the values of each lamb in each treatment.

Different FA profiles were measured in muscle samples from lambs grazing different treatments. The 4H treatment had the highest (*P* < 0.001) proportion of C17:1 and tended (*P* = 0.060) to have reduced C18:0 (Table 3). The 2H treatment had a lower proportion of C18:1 trans-11 (vaccenic acid, VA) compared to the other three treatments (*P* = 0.0002). As compared to the other grazing times, the 2H treatment had lower proportions of C18:2 trans-9, trans-12 (*P* = 0.003), C18:2 cis-9, cis-12 (linoleic acid, LA; *P* = 0.014) and CLA cis-9, trans-11 (*P* = 0.004) than the other treatments, which were not significant different from each other. Lambs with 12-h grazing access had higher (*P* = 0.014) LNA proportion as compared to the 2-h of access, while those with 4- and 8-h pasture access had intermediate levels. Muscle LNA level increased with increased dietary LNA level (Fig. 4a) or decreased dietary LA level (Fig. 4b). For a 100 g rise in dietary LNA, lamb meat LNA increased by 12 g kg^−1^, while for a 100 g rise in dietary LA, lamb meat LNA decreased by 19 g kg^−1^. The greatest (*P* < 0.001; *P* = 0.005) proportions of C20:2 and of C22:0 were separately found in the 4H and 2H treatments. The muscle of the 4H and 12H treatment lambs contained greater (*P* = 0.034) proportions of C20:3 n-3 compared to the 2H and 8H treatment lambs; the C20:4 n-6 was found at a higher proportion (*P* = 0.007) in the muscle of the 4H, 8H and 12H treatment lambs compared to the 2H treatment lambs, and there was no difference between the former three treatments. The 4H treatment tended (*P* = 0.052) to have an increased EPA; it also had the highest percentage of DHA, while the 12H treatment had a lowest proportion of this FA, being present in the following order: 4H > 2H/8H > 12H (*P* = 0.0003). The total saturated FA (SFA) content was greater (*P* = 0.016) in the 2H treatment compared to the 4H treatment, while it was at intermediate levels in the 8H and 12H treatments. The total PUFA was higher for the 4H treatment than the 2H treatment (*P* = 0.005), and there was no time-effect as the daily grazing time increased from 4 h to 12 h. The n-6/n-3 ratio and intramuscular fat (IMF) content did not differ across treatments.

**Table 3:** Fatty acid composition of *longissimus dorsi* muscle of lambs restricted to 2-, 4-, 8-, and 12-h access to pasture.

Item	Treatment^1^				s.e.m.	*P*-value^2^
	2H	4H	8H	12H		
C10:0 (%)	0.16	0.17	0.17	0.20	0.008	0.437
C12:0 (%)	0.22	0.22	0.21	0.20	0.011	0.885
C14:0 (%)	2.68	2.59	2.65	2.51	0.074	0.880
C14:1 (%)	0.10	0.08	0.09	0.11	0.005	0.186
C15:0 (%)	0.38	0.47	0.47	0.50	0.019	0.113
C15:1 (%)	0.12	0.11	0.11	0.11	0.009	0.997
C16:0 (%)	23.12	22.81	22.67	21.59	0.233	0.096
C16:1 (%)	1.56	1.58	1.57	1.62	0.040	0.958
C17:0 (%)	1.18	1.32	1.38	1.34	0.041	0.364
C17:1 (%)	0.12^b^	0.17^a^	0.08^c^	0.09^c^	0.008	<0.001
C18:0 (%)	21.40	19.49	20.07	20.94	0.282	0.060
C18:1 trans-11 (%)	0.42^b^	0.60^a^	0.55^a^	0.58^a^	0.018	0.0002
C18:1cis-9 (%)	40.47	39.72	40.01	39.82	0.334	0.879
C18:2 trans-9 trans-12 (%)	0.42^b^	0.51^a^	0.50^a^	0.47^a^	0.011	0.003
C18:2 cis-9 cis-12 (%)	2.78^b^	3.64^a^	3.47^a^	3.88^a^	0.134	0.014
C18:3 n-6 (%)	0.04	0.05	0.04	0.04	0.003	0.584
C18:3 n-3 (%)	0.74^b^	1.16^ab^	1.10^ab^	1.56^a^	0.098	0.014
CLA cis-9 trans-11 (%)	0.47^b^	0.64^a^	0.72^a^	0.80^a^	0.036	0.004
CLA trans-10 cis-12 (%)	0.12	0.14	0.15	0.19	0.011	0.283
C20:0 (%)	0.18	0.17	0.22	0.21	0.010	0.224
C20:1 (%)	0.08	0.08	0.08	0.10	0.006	0.542
C21:0 (%)	0.38	0.34	0.46	0.41	0.073	0.953
C20:2 (%)	0.12^b^	0.21^a^	0.13^b^	0.12^b^	0.010	<0.001
C20:3 n-6 (%)	0.15	0.10	0.14	0.17	0.010	0.097
C20:3 n-3 (%)	0.02^c^	0.04^b^	0.02^c^	0.08^a^	0.005	0.034
C20:4 n-6 (%)	1.73^b^	2.09^a^	1.98^a^	1.93^a^	0.041	0.007
C20:5 n-3 (%)	0.09	0.13	0.11	0.11	0.002	0.052
C22:0 (%)	0.08^a^	0.05^b^	0.05^b^	0.07^ab^	0.004	0.005
C22:6 n-3 (%)	0.35^b^	0.44^a^	0.31^bc^	0.23^c^	0.021	0.0003
SFA (%)	49.71^a^	47.12^b^	48.13^ab^	48.17^ab^	0.307	0.016
MUFA (%)	43.73	43.60	42.85	42.30	0.282	0.245
PUFA (%)	6.79^b^	8.32^a^	7.78^a^	8.51^a^	0.204	0.005
n-6/n-3	3.74	3.68	3.93	3.79	0.153	0.955
IMF (g kg^−1^ DM)	4.42	3.03	3.33	3.38	0.291	0.350

^a–d^Means within a row with different superscripts differ significantly (*P* < 0.05). ^1^Treatments: 2H = 2-h access to pasture; 4H = 4-h access to pasture; 8H = 8-h access to pasture; 12H = 12-h access to pasture (control). ^2^Probability values for main effects. SFA = saturated fatty acid; MUFA = monounsaturated fatty acid; PUFA = polyunsaturated fatty acid; IMF = intramuscular fat; DM = dry matter; s.e.m. = standard error of mean.

## Discussion

This study investigated the changes in lamb foraging selectivity in response to increasing restriction of the time at pasture and increasing supplementation of forage with concentrate and pasture hay. The primary aim was to determine the influence of the different grazing scenarios on FA composition in the diet and resulting lamb meat.

The feed quality attributes of the pasture and supplementary feeds in this experiment were similar to high-quality alfalfa. The average value of CP in *A. ramosum* was higher than the concentrate supplement, and previous measurements of high-quality alfalfa that had 167–170 g CP kg^−1^ dry matter (DM) at flowering stage^7^. *C. squarrosa* and *C. duriuscula* had moderate fibre contents; their NDF could be comparable to that of dehydrated alfalfa that was cut at the flowing stage (*i.e*. 418 g kg^−1^ DM)^7^. *S. krylovii* and *L. chinensis* had higher ME than other forage species; this is possibly attributed to those gramineae forages containing higher amounts of non-fibre carbohydrate. High CP and ME, as well relatively low fibre content, were directly related to dietary selection and lamb intake of a forage species.

Allowing lambs access to pasture provides a natural source of LNA, and it plays a key-role in the n-3 PUFAs synthesis in meat and milk^8^. In this study, the most significant difference was found in LNA concentration, which was higher (*P* < 0.001) in five individual forage species than in the concentrate supplement. Of those five species, *L. chinensis* had the highest LNA during the growing season, which was comparable to high-quality alfalfa that had 7.5–13.3 g kg^−1^ DM during summer and autumn^9^. Concentrate is generally rich in C18:1 and LA^10^, but C20:3 n-3 or EPA was not detected in the concentrate used in this study. It is important to note, for a grassland with abundant plant species, long-chain fatty acids of C20:3 n-3 and EPA were almost only found in *A. ramosum*, which is a member of *Allium* family. This supports anecdotal observations that Sunite sheep meat from *Allium* dominant desert steppe has high acceptability among consumers, and it is also perceived to have a high nutritional value. In addition to plant species, chemical composition varies due to growing season. The proportions of LNA and LA were higher in autumn compared with the other seaons^9^. In the present trial, LNA and EPA tended to decline but in contrast palmitic and stearic acids trended to increase with developing physiological stages of the forages.

The factors influencing foraging behaviour are highly relevant to better understanding the impacts of grazing on grassland and, therefore, the design of appropriate management schemes. Foraging selectivity of free-grazing livestock is usually affected by plant nutrition, sward structure and vegetation availability^11^. In the present experimental region, the pasture components, *S. krylovii*, *C. squarrosa*, *L. chinensis*, *C. duriuscula* and *A. ramosum*, made up the diet of the lamb. The respective high proportions of *C. squarrosa* and *C. duriuscula*; *L. chinensis* and *C. squarrosa* in the diet of lambs with longer (8–12 h) or shorter (2–4 h) grazing time access are an important finding of this study. Specifically, the proportion of *L. chinensis* decreased with increasing grazing time. The lambs with greater time on pasture grazed from morning to afternoon and had the opportunity to select their desired forages of *C. squarrosa* and *C. duriuscula*, which are lower in fibre over the growing months (Table 1). Animals are generally less selective in the morning after overnight fasting, and grazing selection is generally higher during the afternoon^12^. Additionally, there are more WSC in the grasses in the afternoon^4,13^. *C. squarrosa* is the main plant species in the experimental region, and was highly available to lambs; therefore it was a preferred diet component of lambs for both longer and shorter grazing times. Lambs/kids prefer to browse on fresh, young and palatable forages at pasture^14,15^. The high palatable and nutritional content of *A. ramosum* most likely accounted for it being strongly selected by lambs in July. However, as this sulphide-containing plant matured there was a large reduction in its average consumption of four treatments in mid-August (−50.91%, Fig. 2), although its CP content was still higher than *L. chinensis* (18.58% *vs*. 14.45%, data not shown) in this period. This argument confirmed that changes in preference were likely due to differential changes in the palatability of plant species.

Studies commonly found that herbivores preferentially select plant species relatively high in nutrients^16^. However, it was not the case with *L. chinensis*, as it had a higher CP content than *C. squarrosa* and *C. duriuscula* but the lambs with longer access time at pasture did not focus their grazing on it, even when nutrient levels were highest in July. This is likely attributed to a native physical feature of *L. chinensis* of fine serrations covering the leaves, which likely resulted in poor palatability of *L. chinensis* for the young lambs. The intake rate of the forages has previously been related more with preference than nutrient levels^17^. A previous study demonstrated that as a main plant species in the Songnen plain, *L. chinensis* was virtually neglected by goats when more nutritious species were available^18^. However, under the limited conditions, *L. chinensis* was a suitable substitute. The lambs restricted to 2–4 h access had larger proportions of undesirable *L. chinensis* (with high CP and ME contents), mainly because they had to quickly acquire food in a limited grazing period to optimise nutrient intake, consequently resulting in an average increase in *L. chinensis* consumption, 4.4 times higher than when time at pasture was reduced from 8–12 (8.91% *vs*. 39.4%, on average). The similar effects of higher selectivity in diet were observed in young goats^15,19^. It could be concluded that young animals show a partial preference for palatable forages in most circumstances, but they were remarkably adept at selecting highly nutritious forage when time to access pasture is limited on resource-available grasslands. Our findings on the diet selection of lambs with restricted grazing time lambs suggest that reducing the grazing of animals could be used in grazing management to reduce the damage to the palatable forages in the typical grassland.

The variability in selection over the season was consistent with relative changes in the nutritive quality of the forage species but the palatability was also important. In the case of *A. ramosum*, its selection by lambs peaked in July, and declined with a change in taste or nutrition. It was then replaced with *C. squarrosa*, a lower preference species that made up more than 60% of the diet in August, particularly for the lambs with longer time at pasture. The consumption of *C. squarrosa* declined in September whilst its fibre content increased consistently as the month progressed. In this period of scarcity, the lambs tended to select more *L. chinensis*, particularly in the middle of September when utilization reached its peak. It is highly likely that lambs prefer to select quality forage when feed was limited. *L. chinensis* is a perennial that remained green when other forages were desiccated in mid-September and it had higher selection at this time, consistent with lamb seeking species with a higher nutritive value.

Although *S. krylovii* occupied a certain proportion of the four diets, it was only excessively consumed in July. Across the grazing period, the average selectivity indices of *S. krylovii* were all negative values, indicating a lower usage of it throughout the grazing season relative to its high fibre content. Similar to the present findings, adult sheep also showed negative selectivity indices of *S. krylovii* over the grazing season^20^. The variation in meal to meal preferences may also be partly deterministic in nature according to seasonal changes in forage availability. The decline in consumption of *A. ramosum* corresponds to its disappearance from pasture in September.

The feeding regime as, pasture or concentrate, significantly affected the total n-3 PUFA in lamb meat^21^. We further found that types of meat fat deposits were different depending on the grazing management. In the meat of the 4–12 h grazed lambs there was greater VA, LA, LNA, CLA, AA and PUFA contents compared to the 2 h grazed meat. This is due to higher LNA intake by the lambs that had 4–12 h grazing at pasture than the lambs that grazed 2 h at pasture (data see Table 3). Lambs allowed to graze for 4 h in the afternoon had higher VA, LA, LNA and PUFA in meat than those that grazed for 4 h in the morning^4^. The authors attributed this difference to the sward being relative higher in LNA in the afternoon than in the morning. The linear regression results in this study suggest that amount of LNA accumulated in meat strongly depended on the dietary intake of LNA instead of LA. LNA is the precursor of the endogenous synthesis (through elongation) of EPA and DHA in tissues of the animals^22^. As expected, the meat of 4–12 h grazed lambs in this trial presented greater percentages of EPA and DHA than the 2 h lambs, due to the animals with 2-h grazing had more concentrate supplement which is rich in LA. Therefore, the results of this study suggest that for grazing management, the animals substitute grass hay in place of pasture, especially when they have limited access time to pasture.

Meat from the lambs allocated to 4–12 h grazing had lower (*P* = 0.060) concentrations of stearic acid than the meat from lambs allocated to 2 h of grazing. This result cannot be justified by the stearic acid intake, as this FA intake was present at higher levels in the 4–12 grazed lambs. A previous study^4^ reported a similar finding on restricted 4 h-grazing lamb meat FA profile. The authors mainly attributed this effect to ryegrass containing alkaloids, which could inhibit the biohydrogenation (BH) of PUFA by ruminal bacteria. In the current study however, the variation is possibly attributed to different ruminal pH values^23^ influenced ruminal BH patterns as the lambs allocated 2 h at pasture that consumed a high level of concentrate that therefore had a faster rate of reduction in ruminal pH value^24^. In contrast with the other saturated fatty acids, stearic acid — a saturated fatty acid with 18 carbon atoms — is considered to not have a negative effect towards humans^25^. Research indicated that stearic acid had no effects on serum total cholesterol concentrations and increased intake of stearic acid reduces the risk of cardiovascular disease similar to intake of oleic acid (C18:1)^26^.

It is of unquestionable interest that restricting lambs to graze 4 h induces a greater accumulation of PUFA and lower SFA in meat compared to lambs grazed for 2 h, and the effect was equivalent to a longer (8 h) or whole day grazing management. It has been largely proven that a high SFA intake is associated with an increased risk of chronic diseases in humans^27^. Our data suggest that specific compounds in the diet can be transferred to the meat. For this reason, we considered that, the meat FA profile of 4-h grazed lambs was comparable to the longer grazed lambs because the lambs restricted to 4 h at pasture had a larger consumption of *L. chinensis* than the longer grazed animals, with *L. chinensis* being high in LNA. On the other hand, these lambs had a consumption of 20% *A. ramosum*, equally to the longer (8–12 h) grazed animals. *A. ramosum* is usually considered as high-quality forage, with a higher component of EPA. Importantly, the lambs restricted to graze 4 h per day can improve DHA accumulation in meat. This is relevant to both feed composition and walking activity, as the balance between CP and carbohydrate in the diet governs ruminal functionality^28^. We previously proposed that the lambs restricted to 4 h or less at pasture could reduce energy requirement for walking activities, and increase ME available for their growth through a reduction of mean travelling distance during grazing by 43% compared to 8–12 h grazing period.

For humans more than 95% of the intake of DHA and EPA is directly found in food that is consumed, and has beneficial effects on reducing cardiovascular risk, regulating the inflammatory response and promoting neurodevelopment^29^. In addition, the meat from the four groups of animals presented similar n-6/n-3 values. UK Department of Health^30^ recommended a dietary n-6:n-3 ratio of 4 or less for human consumption in order to reduce the risk of cardiovascular disease. In the meat samples tested in the current study, this ratio was well below the value of 4.

## Conclusion

Our findings suggest young lambs with a restricted access of 4 h per day at pasture have the strong ability to alter their grazing behaviour to modify forage selection. The animals preferred to browse on more palatable forage with a lower fibre content, while demonstrating a partial preference to high protein forages. *L. chinensis* was more preferentially selected by lambs grazing pasture for shorter periods than longer periods. Ultimately, this optimised nutrient intake when given a shorter time at pasture. This modified dietary pattern resulted in a high LNA intake by the lambs allowed to graze for at least 4 h per day. LNA and its elongation products, as well as total PUFA accumulation in tissue, depended on the LNA level in diet. Therefore, allowing lambs to graze for at least 4 h per day resulted in a meat FA profile richer in health-promoting FAs. In particular, the highest percentage of DHA was observed in the meat from lambs which grazed for 4-h versus 8- and 12-h. It may be concluded that, in grassland systems similar to those in the present study, it is most beneficial to limit lambs to graze 4-h (while supplementing with hay and concentrate) rather than grazing longer time periods or for the whole day, thus resulting in a healthier meat FA profile in humans and the potential for better pasture management.

## Material and Methods

### Study site

The experiment was conducted over a 99-day period from early July to late September 2011. This period of time corresponded with high pasture availability, in an experimental farm located at Maodeng in eastern Xilinguole (116° 30′ E, 44°49′ N; alt. 1200 m a.s.l.), Inner Mongolia, China. The area has a semi-arid continental monsoon climate with an average annual precipitation of 350 mm, occurring mostly between July and August. A yearly average temperature of 2.0 °C, the mean temperature is 18−21 °C in June-August. The non-frost period is 90–120 days from May to September. The native vegetation is generally composed of *S. krylovii*, *L. chinensis*, *C. squarrosa*, *C. duriuscula* and *A. ramosum*, which represents 54–57%, 21–31%, 10–15%, 6–10% and 0–4%, respectively, of the botanical composition on offer in July-September and provides the bulk of the food for grazing sheep. The most abundant forage is in August, with annual species of *Salsola collina* and *Chenopodium album* also present, but with a scattered and patchy distribution. The quantity and quality of this pasture have been stated by our previous work^3^. Ujumuqin sheep, the most important cross breed for local sheep production, is highly regarded by herders for its high meat quality, especially in lambs. The lambs are born in March and are sold by September. In the region, sheep are traditionally grazed all year, except for the 40–60 days of closed-grazing imposed by the government in early spring.

### Animals and management

All experimental procedures, including animal ethics, were approved by the Office of Beijing Veterinarians (the Agriculture Ministry, Beijing, China), and all methods were performed in accordance with the relevant guidelines and regulations^31^. Thirty-two, castrated and weaned male, Ujumuqin lambs, with similar body weight (21.86 ± 0.38 kg) and age (120 ± 15 days), were randomly assigned to one of the following four equal treatments: (i) 2-h access to pasture (2H), (ii) 4-h access to pasture (4H), (iii) 8-h access to pasture (8H), and (iv) 12-h access to pasture (12H; control). A total of 30 hectares of pasture were fenced off into three plots, the plots of 10 ha each were divided into four equal paddocks, giving a total of 12 paddocks of 2.5 ha each to avoid the possible bias on pasture availability between treatments. The lambs began to access pasture at 6:00 and were removed at 8:00, 10:00, 14:00 and 18:00 for 2H, 4H, 8H and 12H treatments, respectively. At the end of the set time allowed at pasture, lambs for each treatment were separately housed in 32 individual pens and received supplements of concentrate (consisting of 63% chopped maize, 10% wheat bran, 10% soybean meal, 8% cottonseed meal, 8% rapeseed meal and 1% premix) and *Chrysopogon aciculatus* hay. The concentrate was offered separately at 18:30 every day within individual pens; and the amount fed per sheep, on a DM basis, was 383 g in the first two months and 425 g in the last month for the 2H treatment; 255 g in the first two months and 290 g in the last month for the 4H treatment; 215 g in the first two months and 255 g in the last month for the 8H treatment; and 110 g in the first two months and 150 g in the last month for the 12H treatment. The grass hay was fed as free access from 6:00 to 21:00 and was removed from troughs after 21:00 each day; and it was adjusted daily on the basis of the previous day’s intake, allowing refusals of 20%. The amounts of concentrate and grass hay offered to lambs were determined to obtain an average target energy and protein intakes of 13.6 MJ day^−1^ and 181 g DM day^−1^, respectively. The average amounts of individual feed and nutrient intakes of lambs, as well as herbage allowance, are given in Table 4. Pasture DM intake was estimated for each treatment using the alkanes method in the middle of July, August and September; detailed information for this procedure and estimation of results are reported in our previous study^3^.

**Table 4:** Amounts of concentrate, grass hay and pasture consumed by lambs and herbage allowance of pasture in Inner Mongolia, China.

Item	Treatment^1^			
	2H	4H	8H	12H
Concentrate^2^ (kg day^−1^)	0.40	0.27	0.23	0.12
Grass hay (kg day^−1^)	0.34	0.25	0.13	0
Pasture (kg DM day^−1^)	0.62	0.89	0.99	1.24
Total DM intake (kg day^−1^)	1.36	1.41	1.35	1.36
CP intake (g DM day^−1^)	178	185	181	181
ME intake (MJ day^−1^)	13.8	14.1	13.4	13.1
NDF intake (g DM day^−1^)	645	762	750	836
Herbage allowance (kg DM ha^−1^)	992	953	951	932

^1^Treatments: 2H = 2-h access to pasture; 4H = 4-h access to pasture; 8H = 8-h access to pasture; 12H = 12-h access to pasture (control). ^2^The concentrate consisted of 63% chopped maize, 10% wheat bran, 10% soybean meal, 8% cottonseed meal, 8% rapeseed meal and 1% premix. The premix contained (per kg) 10500 IU vitamin A, 2110 IU vitamin D_3_, 43 mg vitamin E, 40 mg Mn, 32 mg Fe, 95 mg Zn, and 16 mg Cu. DM = dry matter; CP = crude protein; ME = metabolizable energy; NDF = neutral detergent fibre.

Before the start of the experiment, the animals were adapted to the experimental conditions over a 12-d period, in which they were introduced to the pasture for the time established for each treatment and they received set amounts of concentrate and grass hay. The animals were given *ad libitum* access to water and salt blocks throughout the experimental period.

### Estimation of diet composition

The N-alkanes method was used to estimate diet composition in the middle of July, August and September. At each of the three time points, six lambs from each treatment were dosed indoors with a dotriacontane pellet twice daily before grazing and after grazing for a 12-d period. From day six post-dosing, faecal samples were collected from each lamb twice daily. The faecal samples from each lamb on each sampling day were immediately oven-dried at 65 °C for 48 h, six days worth of faecal samples were pooled for each lamb after each sampling period, and milled through a 1-mm sieve for subsequent n-alkanes analysis. The proportions of forage components consumed by the animals were estimated based on n-alkanes patterns, using a non-negative least squares procedure in the computer package ‘Eatwhat’^32^.

To determine the plant species and parts consumed by the lambs, the foraging behaviour of the lambs was monitored by visual observation in the middle of July, August and September. During the observation periods, lambs were observed for a period of 30 min, in which samples of individual plants species were plucked to represent the average bite consumption. Samples were collected manually from 9 random sampling sites within each plot. For each month, 27 individual herbage samples (0.2–120 g per sample) were pooled together to obtain three subsamples. The bite-consumption herbage samples were dried in a forced-air oven at 65 °C to a constant mass. Dried samples were measured for DM, and then ground with a mill (CM200, Retsch GmbH, Haan, Germany) and passed through a 1-mm screen for subsequent n-alkane and FA analysis.

A selectivity index was also calculated, defined as the proportion of a plant type in the diet relative to its proportion in the pasture period. This was quantified by calculating selectivity indices (D) for each feed item using Jacobs’^19,33^ selectivity index (D varied from 0 to 1 for positive selection and from 0 to −1 for negative selection). We chose Jacobs’ index as it is independent of the relative feed abundance, and directly reflects sheep selection and the impact on a given feed item in the environment.

### Slaughtering and muscle sampling

At the end of the experimental period, all lambs were fasted overnight and transported to a commercial abattoir. The lambs were slaughtered by exsanguination from the jugular vein. Within 45 min of slaughter, the *longissimus dorsi* was excised at the level of 10–13th rib from the left side of the carcass, wrapped in aluminium foil, vacuum-packaged and frozen at −80 °C for fat and FA analysis.

### Chemical analyses

N-alkane content of individual feeds were measured according the modified procedure^34^. Feed DM content was determined according to AOAC^35^; CP was determined with a Kjeldahl analyser (Kjeltec 2300, Hoganas, Sweden), and NDF was determined by the filter bag technique (ANKOM 2000, Fairport, NY). ME in feed was calculated using the equation^36^: $ME(Mcal\,k{g}^{-1}DM)=1.01\times 0.04409TDN-0.45$. IMF was measured with a fat analyser (ANKOM XT 15, Fairport, NY), using 200 mg of freeze dried and grounded *longissimus dorsi* sample.

Fatty acid compositions of feed samples and freeze dried *longissimus dorsi* samples were quantified us Agilent 6890 N Gas Chromatograph (GC) (Agilent technologies Inc., Wilmington, DE). Anhydrous chloroform/methanol (1:10, v/v) was used for the methylation of the FAs and henedecanoic acid methyl ester (C11:0) was used as internal standard (1 mg ml^−1^). The oven temperature was set at 130 °C for 1 min then was increased up to 230 °C at a rate of 4 °C min^−1^. The injector and detector (FID) temperatures were set at 270 and 280 °C respectively, and the helium flow ratio was 1 ml min^−1^. An automatic split injector as used with a ratio 50:1 split. The individual FA peaks were identified by comparison of retention times with those of known mixtures of standard FA (37 component FAME mixture) run under the same operating conditions.

### Statistical analyses

The proportions of the forage components consumed and FA intakes by lambs in each treatment from July to September were analysed using repeated measures in the MIXED procedure of SAS, with a model considering the forage species, the month of sampling and their interaction as fixed factors, while the individual plot and sheep were considered as random factors. When this model was used to analyse the chemical composition of forage species, the individual forage samples were considered as random factors. A two-sample t-test for means was performed to examine the difference between plant species and concentrate and grass hay. Muscle FA composition was analysed by ANOVA as a completely randomized design, with a model that included treatment effects and experimental error. Individual animals were considered as experimental units. When the ANOVA was significant (*P* < 0.05), means were separately by Duncan’s multiple range test. Simple linear regression analysis was used to evaluate the relationships between dietary LNA and LA intakes and muscle LNA levels of lambs.

### Data availability

All data generated or analysed during this study are included in this published article.
